# Spontaneous regression of colorectal liver metastasis with coexisting IgG4-rich inflammatory pseudotumor-like changes: a case report

**DOI:** 10.1007/s12328-026-02387-0

**Published:** 2026-06-28

**Authors:** Shoichiro Mizukami, Tatsuya Shonaka, Tomoki Takizawa, Katsuro Enomoto, Hiroyuki Takahashi, Koji Imai, Yuki Kamikokura, Sayaka Yuzawa, Mishie Tanino, Hideki Yokoo

**Affiliations:** 1 Division of Gastroenterological and Transplant Surgery, Department of Surgery, Asahikawa Medical University, 2-1-1-1, Midorigaoka-Higashi, Asahikawa, Hokkaido 078-8510 Japan; 2 Department of Diagnostic Pathology, Asahikawa Medical University Hospital, 2-1-1-1, Midorigaoka Higashi, Asahikawa, Hokkaido 078-8510 Japan

**Keywords:** Colorectal liver metastases, IgG4-related disease, Inflammatory pseudotumor, Spontaneous regression

## Abstract

Spontaneous regression of colorectal liver metastasis (CRLM) is an extremely rare phenomenon with only a few reported cases. However, the underlying mechanism remains unclear. Infection-triggered immune reactivation and inflammation have also been proposed to be potential contributors. IgG4-related disease (IgG4-RD) is a systemic fibroinflammatory condition characterized by IgG4-positive plasma cell infiltration and fibrosis, and rarely manifests in the liver as an inflammatory pseudotumor (IPT). We herein report a rare case of a 71-year-old man with possible spontaneous regression of CRLM accompanied by coexisting IgG4-rich IPT-like changes. Eight months after the laparoscopic Hartmann’s operation for rectal cancer, imaging revealed hepatic lesions in segments S5 and S7, which were diagnosed as CRLM. Partial hepatectomy revealed necrotic adenocarcinoma in the S7 lesion, with IgG4-rich IPT-like inflammatory changes. Postoperative evaluation revealed markedly elevated serum IgG4 levels and IgG4-positive plasma cell infiltration in the mediastinal lymph nodes, fulfilling the diagnostic criteria for systemic IgG4-RD. Twenty months postoperatively, the patient remained recurrence-free without adjuvant chemotherapy or steroid therapy. This case report describes the coexistence of spontaneous tumor regression and IgG4-related hepatic IPT within the same lesion and provides insight into the clinicopathological spectrum linking tumor regression and IgG4-RD.

## Introduction

Spontaneous regression of cancer is defined as the complete disappearance of a malignant tumor in the absence of any therapy capable of inducing tumor regression [1, 2]. It is an extremely rare phenomenon, with only a few reported cases in colorectal cancer, particularly in colorectal liver metastasis (CRLM). The underlying mechanisms remain unclear; however, infection- or necrosis-triggered immune reactivation, alterations in the tumor microenvironment, and inflammatory responses have been suggested as contributing factors [3].

In the present case, spontaneous regression of the CRLM was confirmed pathologically. The regressed lesion showed dense fibrosis and chronic inflammatory cell infiltration, with numerous IgG4-positive plasma cells, consistent with an inflammatory and fibrotic background resembling IgG4-related hepatic inflammatory pseudotumor (IPT). IgG4-related disease (IgG4-RD) is a systemic fibroinflammatory disorder that may occasionally involve the liver as an IPT and has been reported to coexist with various malignancies, possibly as a result of abnormal immune responses to tumor-associated antigens [4, 5]. Herein, we report a rare resected case of spontaneously regressed CRLM associated with IgG4-rich IPT-like changes and discuss its clinicopathological features.

## Case report

A 71-year-old man with a medical history of hypertension was referred to our institution. His regular medications included lansoprazole and azilsartan. He consumed approximately 500 mL of beer daily and had smoked 20 cigarettes per day for 45 years. Eight months earlier, he had undergone a laparoscopic Hartmann operation for rectal cancer. Postoperative histopathological examination revealed moderately differentiated adenocarcinoma, classified as pT3N0M0, Stage IIa (R0 resection, Cur A), with microsatellite stability, RAS and BRAF wild-type, and human epidermal growth factor receptor 2-negative status [6]. The patient did not receive adjuvant chemotherapy and was followed up without additional treatment.

**Fig. 1:**
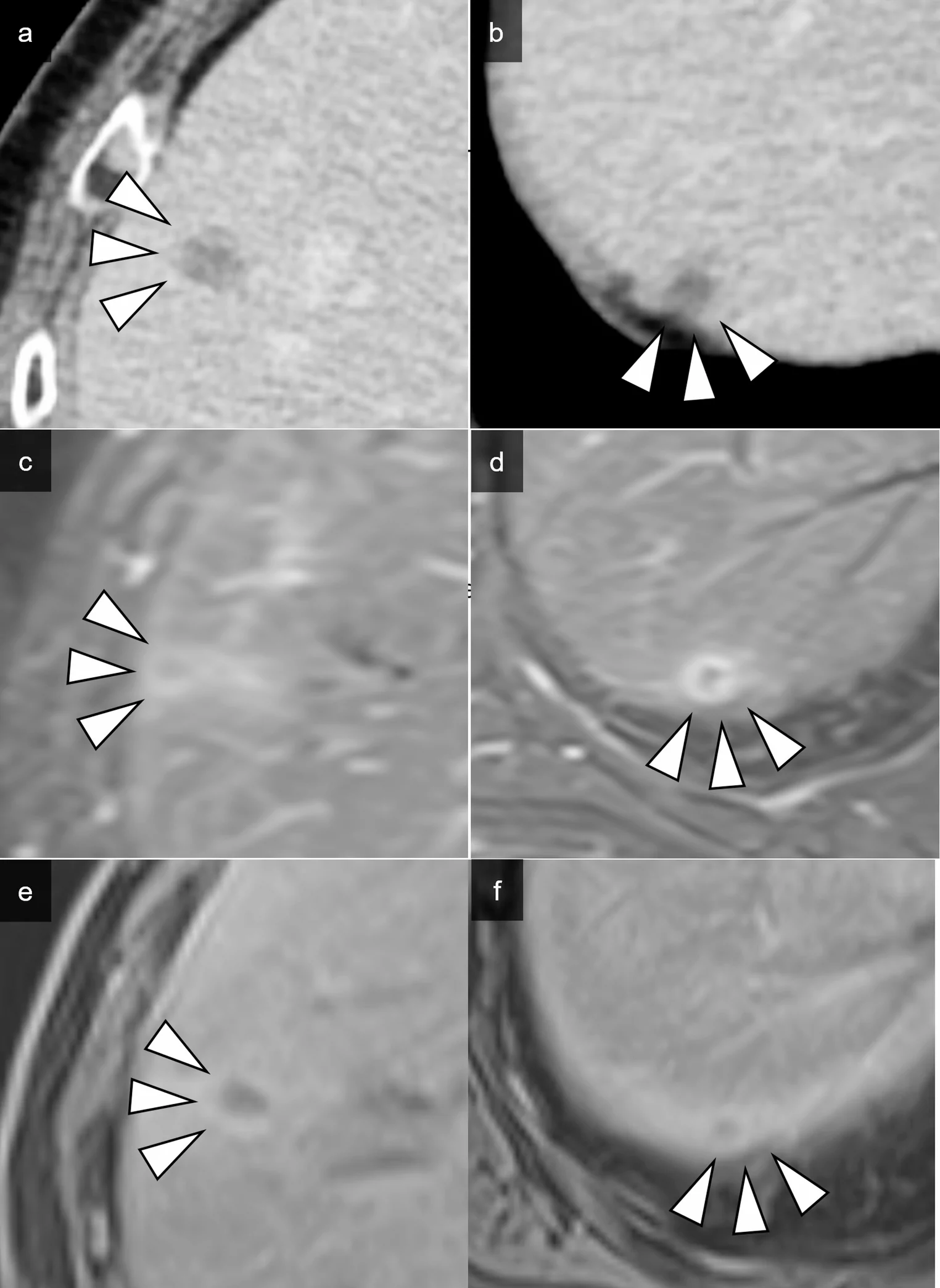
Dynamic CT and EOB-MRI findings of hepatic lesions suggestive of CRLM. **a**, **b** Arterial phase of dynamic CT: A hypodense lesion measuring 11 × 10 mm is observed in the hepatic segment S5. The internal nodular enhancement is obscure, with faint perilesional enhancement noted in the arterial phase (white arrowhead). **c**, **d** T2WI TSE FS of EOB-MRI. **e**, **f** Early phase of EOB-MRI: Round lesions measuring 11 mm in segment S5 and 6 mm in segment S7 are identified. Both lesions exhibit mild hyperintensity on T2WI. Ring-like peripheral enhancement is observed in the early dynamic phase (white arrowhead)

**Fig. 2:**
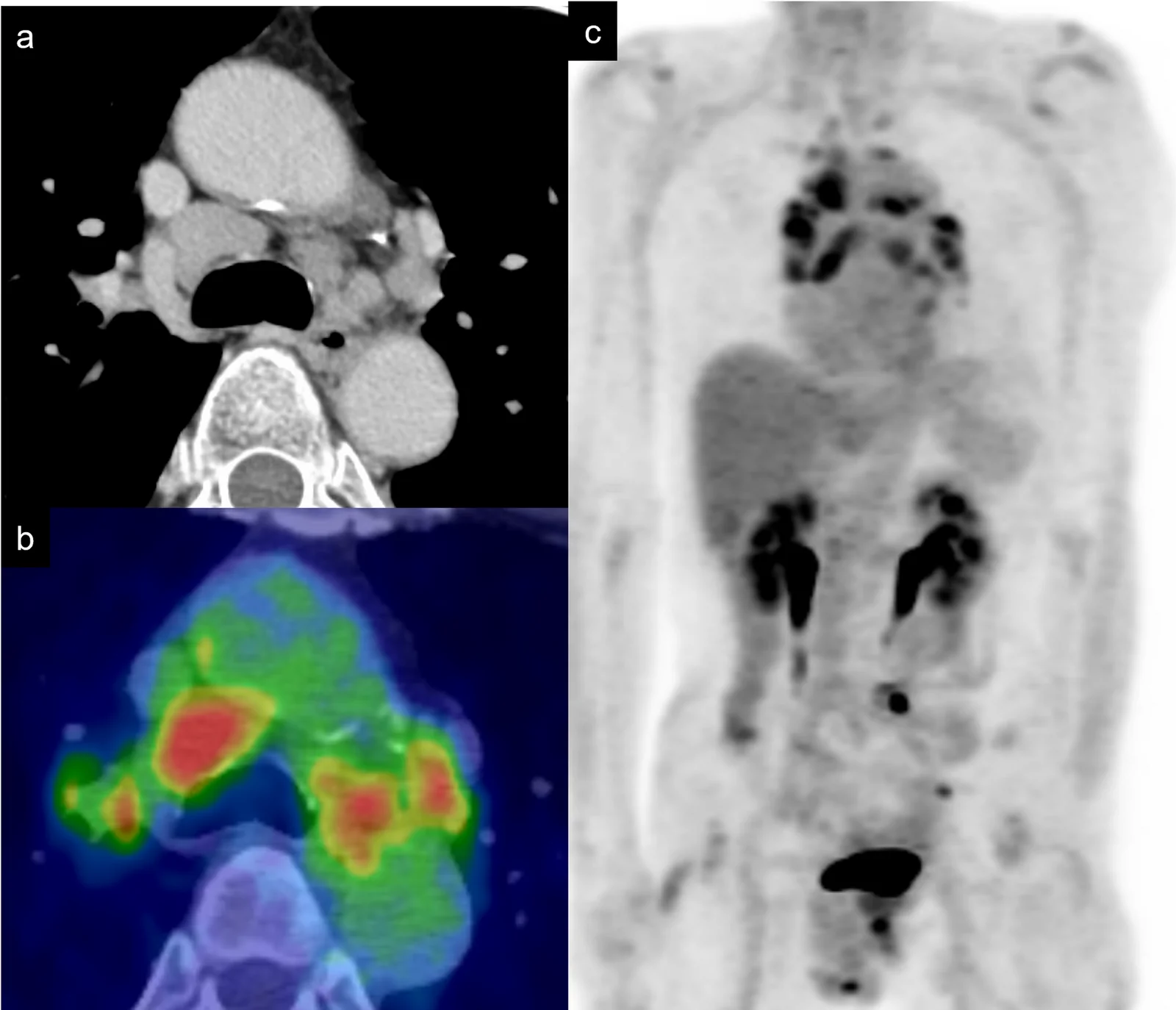
Mediastinal CT and PET emission tomography-CT findings. **a** Mediastinal CT and **b** PET–CT show enlargement and FDG accumulation in the mediastinal and hilar lymph nodes (maximum standardized uptake value, 7.0), interpreted as reactive lymphadenopathy associated with chronic obstructive pulmonary disease. **c** No abnormal FDG uptake is detected in the liver (including the S5 and S7 lesions), pancreas, spleen, adrenal glands, or kidneys

Eight months postoperatively, contrast-enhanced computed tomography (CT) revealed two hypodense hepatic lesions in segments S5 and S7. The serum tumor marker levels were within normal limits (cancer antigen 19 − 9:5 U/mL; carcinoembryonic antigen test: 2.1 ng/mL). The patient did not have a fever before hepatectomy, and the C-reactive protein level was mildly elevated at 1.45 mg/dL. Serum IgG and IgG4 levels were not measured pre-hepatectomy. Dynamic CT revealed an 11-mm hypodense mass in segment S5 and a 6-mm lesion in segment S7. The lesions showed poorly defined internal enhancement, and in the arterial phase, faint perilesional enhancement was observed in the surrounding liver parenchyma, suggesting CRLM (Fig. 1a, b). No significant bile duct dilatation or strictures were observed. Mediastinal CT and positron emission tomography (PET)-CT revealed enlargement and fluorodeoxyglucose (FDG) accumulation in mediastinal and hilar lymph nodes, with a maximum standardized uptake value of 7.0. These findings were interpreted as reactive lymphadenopathy associated with chronic obstructive pulmonary disease (Fig. 2). No abnormal FDG uptake was observed in the liver, pancreas, spleen, adrenal glands, or kidneys. Gadoxetate disodium-enhanced magnetic resonance imaging (MRI) revealed mild T2 hyperintensity in lesions S5 and S7 and diffusion restriction. Ring-like peripheral enhancement was observed in the early phase with reduced uptake in the liver phase (Fig. 1c–f). Based on the clinical course and imaging findings, CRLM was diagnosed, and the patient underwent partial hepatectomy of segments S5 and S7. The patient was discharged without any complications on postoperative day 15.

**Fig. 3:**
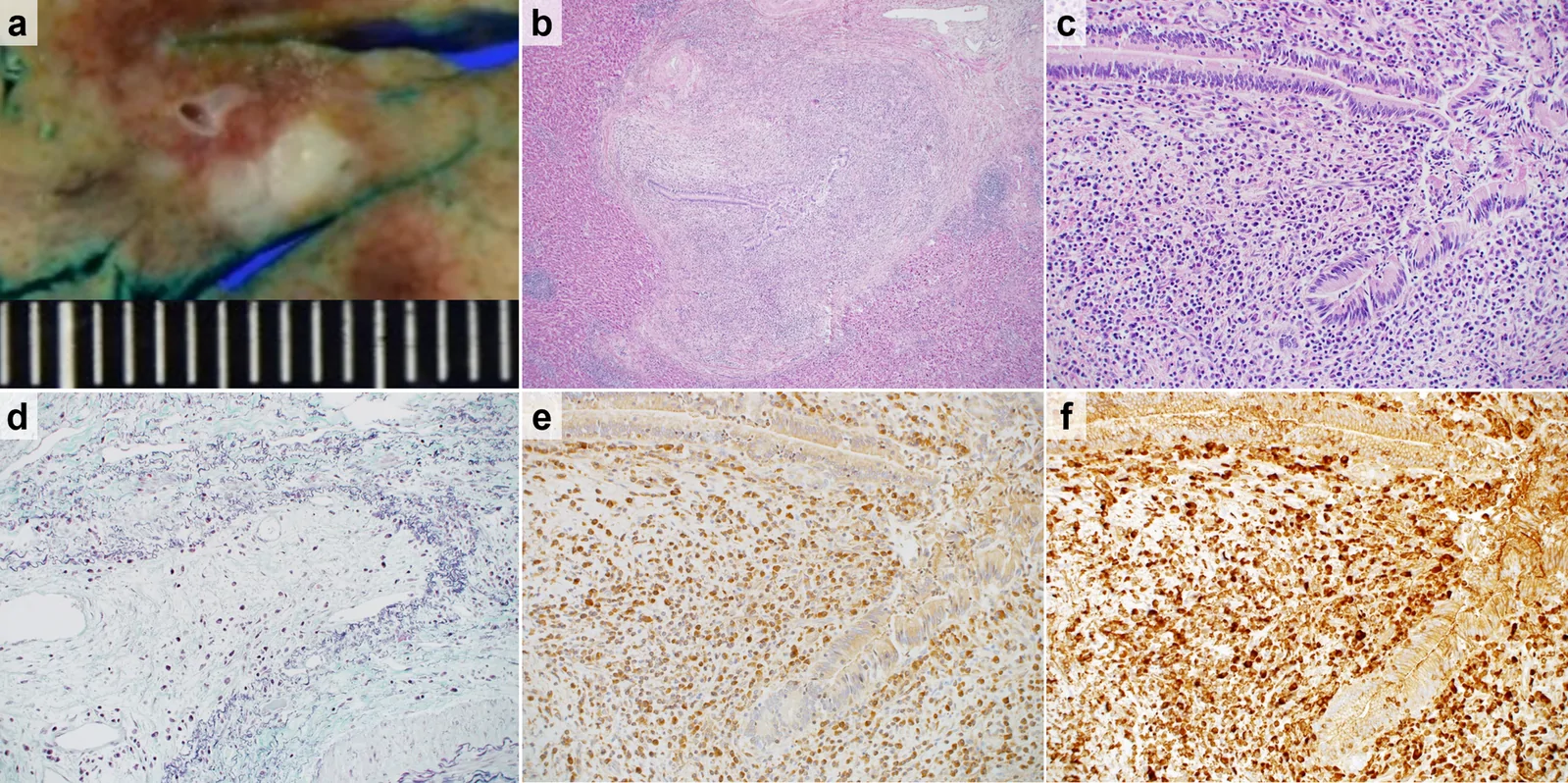
Macroscopic and histopathological findings of the hepatic lesion in segment S5. **a** Gross appearance: A solid grayish-white mass measuring 5 × 5 mm is identified in segment S5 of the liver. **b** H&E, ×40; **c** H&E, ×100: The lesion shows moderate periductal lymphocytic infiltration within fibrotic stroma. Viable carcinoma cells are not observed. **d** Elastica–Masson, ×100: Venous fibrosis with dense infiltration of plasma cells in the vessel wall, consistent with obliterative phlebitis. **e** IgG, ×100; **f** IgG4, ×100: Numerous IgG and IgG4-positive plasma cells are present (approximately 300 cells per high-power field), and the IgG4/IgG ratio exceeded 0.40, findings consistent with IgG4-related hepatic IPT

**Fig. 4:**
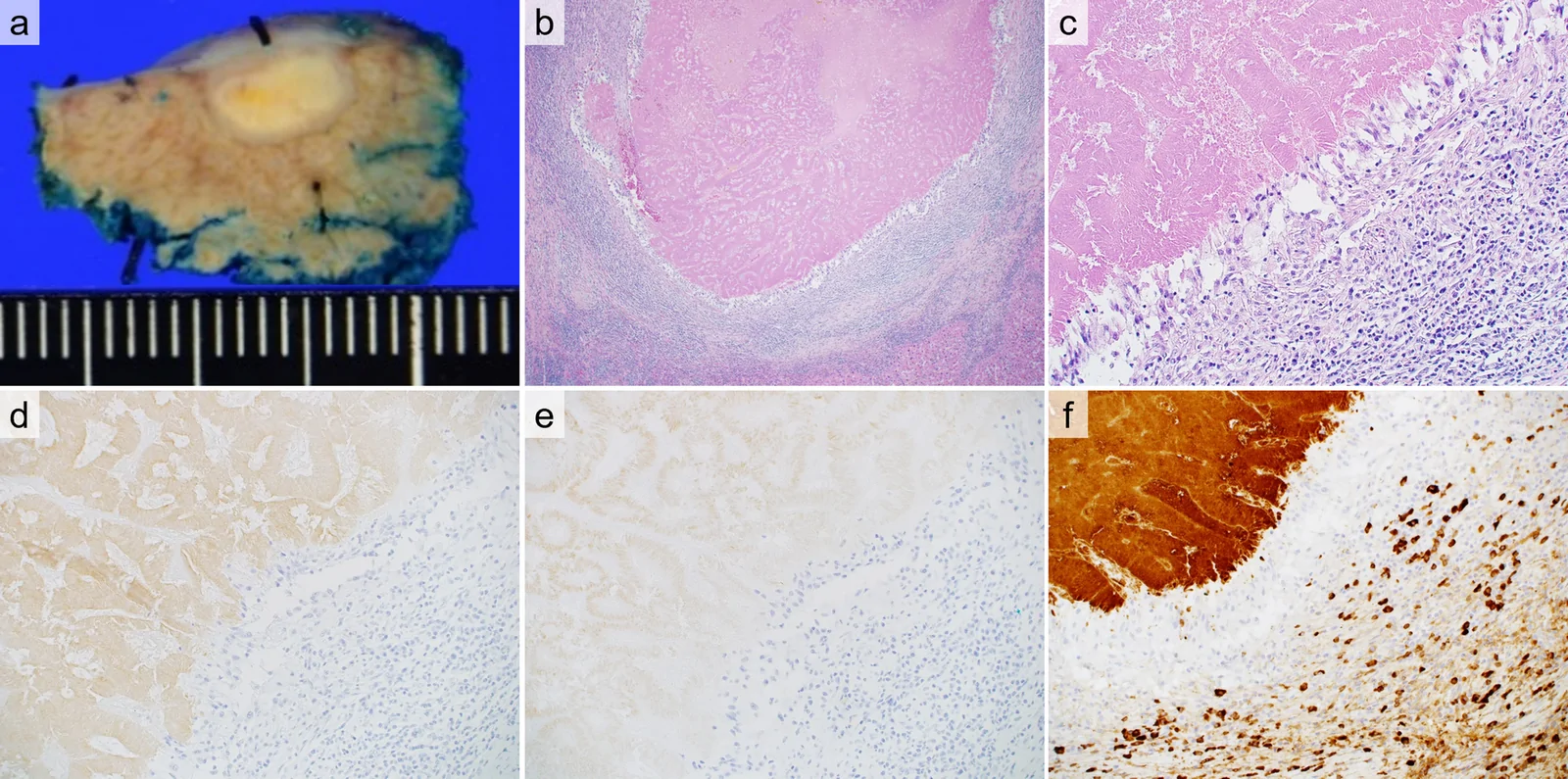
Macroscopic and histopathological findings of the hepatic lesion in segment S7. **a** Gross appearance: A solid grayish-white mass measuring 6 × 6 mm is observed in segment S7 of the liver. **b** H&E, ×40; **c** H&E, ×100: The central area of the lesion shows degeneration and necrosis containing ghost-like adenocarcinoma cells. Surrounding the necrotic region, a dense inflammatory infiltrate composed predominantly of lymphocytes with scattered plasma cells is observed within the fibrotic stroma. The surgical margins were negative. **d** CDX2, ×100; **e** AE1/AE3, ×100: Weak cytoplasmic positivity is noted in ghost-like adenocarcinoma cells, but nuclear staining is absent, indicating loss of viability and consistency with tumor necrosis. **f** IgG4, ×100: Approximately 50 IgG4-positive plasma cells per high-power field are detected, with an IgG4/IgG ratio exceeding 0.40. Similar to the S5 lesion, these findings are consistent with the IgG4-rich IPT-like inflammatory changes surrounding necrotic adenocarcinoma

Gross examination of the resected specimens revealed well-demarcated, solid, grayish-white lesions measuring 5 × 5 mm in segment S5 and 6 × 6 mm in segment S7 of the liver. Histologically, the S5 lesion showed moderate periductal lymphocytic infiltration within the fibrous stroma, along with venous fibrosis and prominent plasma cell infiltration, suggesting obliterative phlebitis. Numerous IgG- and IgG4-positive plasma cells (approximately 300 cells per high-power field [HPF]) were observed, with an IgG4/IgG ratio > 0.40. These findings were consistent with lymphoplasmacytic-type IgG4-related hepatic IPT (Fig. 3). No viable carcinoma was identified in the S5 lesion. In contrast, the S7 lesion showed necrotic glandular structures in the central area, suggestive of metastatic adenocarcinoma. Immunohistochemistry revealed weak cytoplasmic positivity for CDX2 and AE1/AE3 without nuclear staining, and the morphological features were consistent with those of nonviable tumor cells. The surrounding tissue showed fibrosis and inflammatory cell infiltration, with approximately 50 IgG4-positive plasma cells/HPF, indicating IPT-like inflammatory changes associated with the tumor. Taken together, these findings suggest that the S5 lesion represents IgG4-related hepatic IPT, whereas the S7 lesion is consistent with metastatic adenocarcinoma showing extensive necrosis accompanied by IgG4-related IPT-like inflammatory changes, raising the possibility of spontaneous tumor regression in the S7 lesion (Fig. 4).

**Fig. 5:**
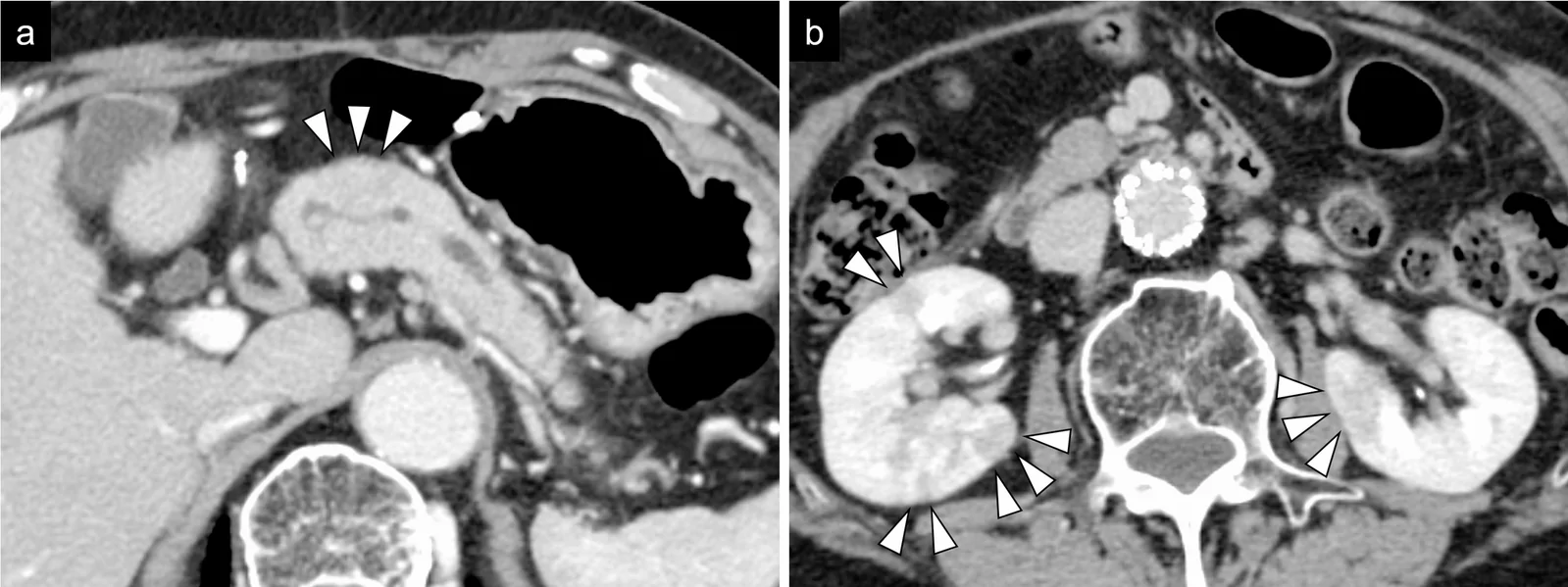
Dynamic CT findings suggestive of IgG4-RD involvement. **a** Diffuse enlargement of the pancreas with mild dilatation of the main pancreatic duct and a nodular change in the pancreatic body (white arrowhead) is observed. **b** Multiple focal areas of poor enhancement with mild cortical atrophy are observed in both kidneys (white arrowhead)

One month post-hepatectomy, serum immunoglobulins were measured for the first time, revealing markedly elevated levels: total IgG, 3284.8 mg/dL (reference range: 861.0–1747 mg/dL); and IgG4, 1530 mg/dL (reference range: 11–121 mg/dL). Postoperative imaging also revealed diffuse enlargement of the pancreas with mild dilatation of the main pancreatic duct and nodular change in the pancreatic body, as well as multiple focal areas of poor enhancement with mild cortical atrophy in both kidneys (Fig. 5). In addition, FDG-avid mediastinal and hilar lymphadenopathy, previously interpreted as reactive changes on PET–CT, was noted (Fig. 2). Taken together, these findings suggest possible systemic IgG4-related involvement.

**Fig. 6:**
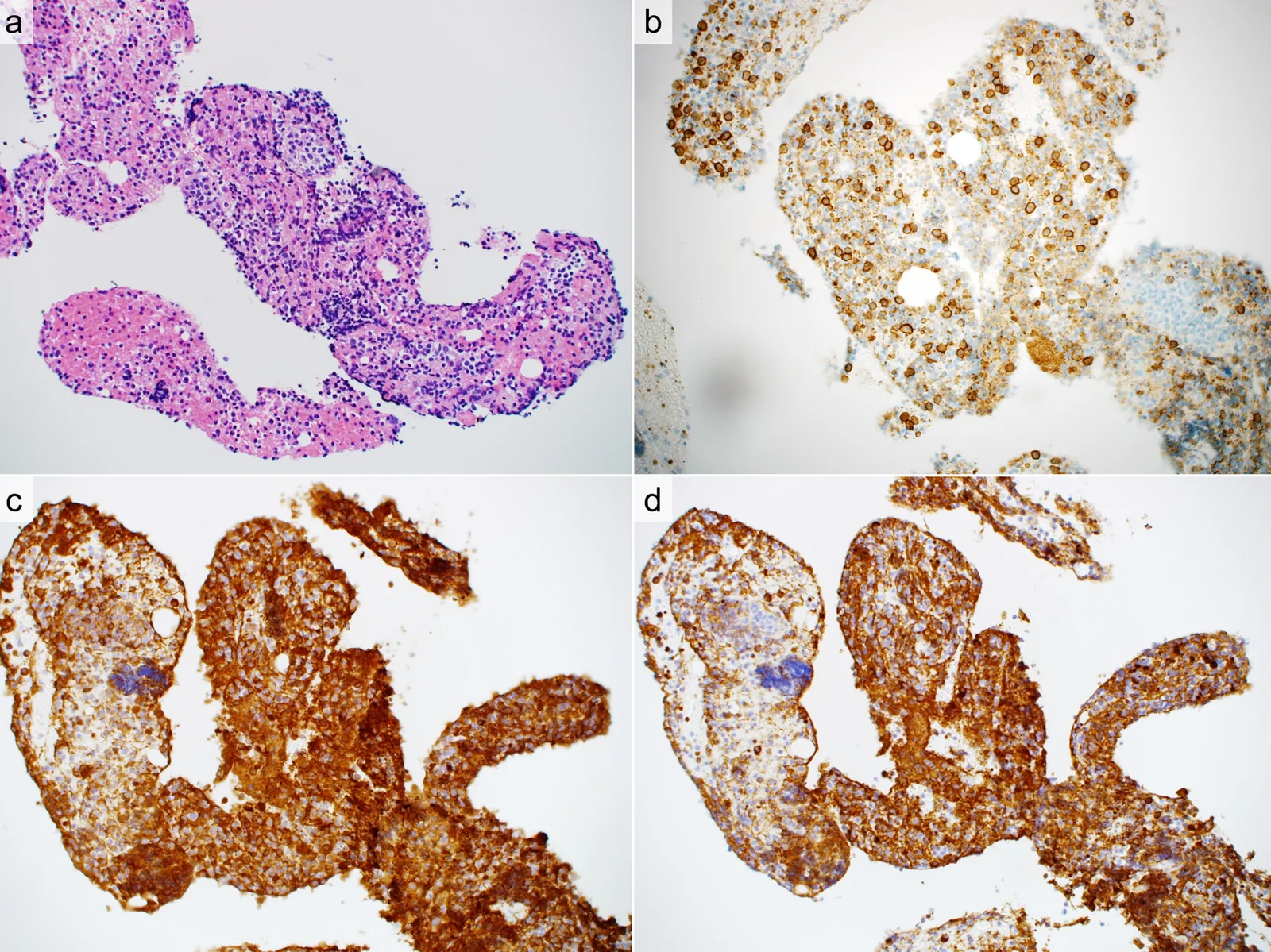
Histopathological findings of TBNA in mediastinal lymph nodes. **a** H&E, ×200: A small amount of lymphoid tissue was obtained and mixed with red blood cells, fibrin, and inflammatory cells. No evidence of fibrosis, obliterative phlebitis, or epithelioid granulomas was observed. **b** CD138, ×200: Patchy infiltration of CD138-positive plasma cells is noted. **c** IgG, ×200; **d** IgG4, ×200: Numerous IgG- and IgG4-positive plasma cells were observed. The IgG4/IgG ratio exceeded 0.40, with more than 100 IgG4-positive cells per high-power field, supporting the diagnosis of IgG4-RD

Three months after hepatectomy, endobronchial ultrasound-guided transbronchial needle aspiration (EBUS-TBNA) was performed to evaluate mediastinal and hilar lymphadenopathy further. Histological examination of the sampled lymph nodes showed dense IgG4-positive plasma cell infiltration, consistent with IgG4-related respiratory disease (Fig. 6). Based on these clinical, radiological, and histopathological findings, the diagnosis of IgG4-RD was established according to the 2020 revised comprehensive diagnostic criteria [7]. The fulfilled criteria and corresponding findings are summarized in Table 1.

**Table 1:** Fulfilled items of the 2020 revised comprehensive diagnostic criteria for IgG4-related disease in the present case

The 2020 revised comprehensive diagnostic criteria for IgG4-RD	Our case
[Item 1] Clinical and radiological features	
One or more organs show diffuse or localized swelling or a mass or nodule characteristic of IgG4-RD. In single organ involvement, lymph node swelling is omitted.	Fulfilled: Diffuse pancreatic enlargement with mild main pancreatic duct dilatation, nodular change in the pancreatic body, multiple renal low-enhancement areas with mild cortical atrophy, and mediastinal/hilar lymphadenopathy were observed.
[Item 2] Serological diagnosis	
Serum IgG4 levels greater than 135 mg/dl.	Fulfilled: Serum IgG4 level was markedly elevated: 1530 mg/dL.
[Item 3] Pathological diagnosis	
Positivity for two of the following three criteria:	Fulfilled: Dense lymphoplasmacytic infiltration with fibrosis, IgG4-positive plasma cell infiltration with an IgG4/IgG ratio > 40%, and obliterative phlebitis/storiform fibrosis were observed in the hepatic lesion. IgG4-positive plasma cell infiltration was also confirmed in mediastinal lymph nodes by EBUS-TBNA.
1) Dense lymphocyte and plasma cell infiltration with fibrosis.	
2) Ratio of IgG4-positive plasma cells /IgG-positive cells greater than 40% and the number of IgG4-positive plasma cells greater than 10 per high powered field	
3) Typical tissue fibrosis, particularly storiform fibrosis, or obliterative phlebitis	
Diagnosis	
Definite: 1) + 2) + 3)	Definite IgG4-RD
Probable: 1) + 3):	
Possible: 1) + 2)	

**Fig. 7:**
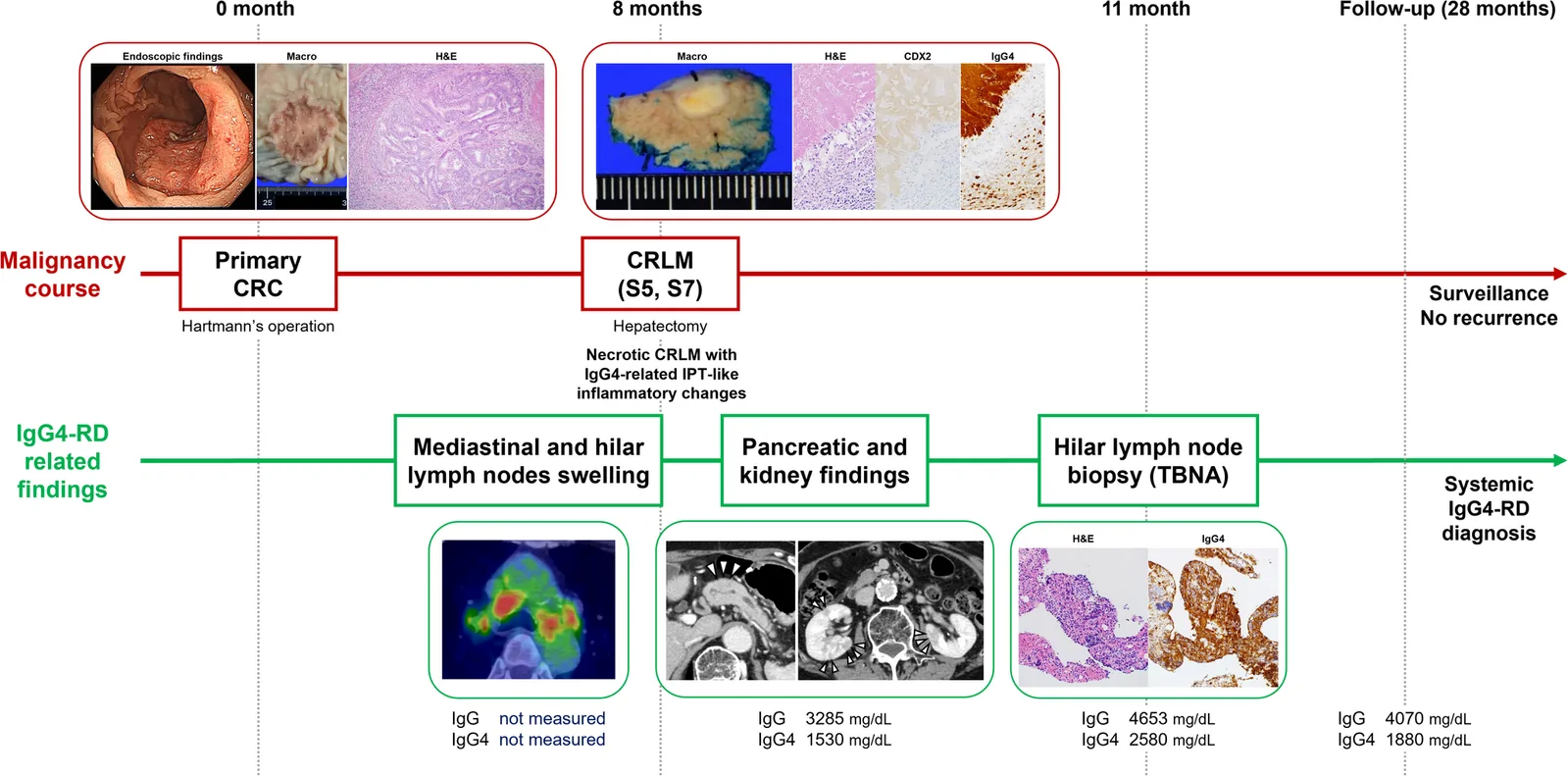
Clinical course of the present case. The upper red timeline shows the malignant disease course, whereas the lower green timeline shows IgG4-RD–related systemic findings. Eight months after Hartmann’s operation for primary colorectal cancer, liver lesions in segments 5 and 7 were resected as suspected colorectal liver metastases. Histopathological examination revealed necrotic adenocarcinoma accompanied by IgG4-related inflammatory pseudotumor-like changes in the S7 lesion. Mediastinal and hilar lymphadenopathy was present before hepatectomy, but serum IgG4 was not measured at that time. Subsequent workup demonstrated pancreatic and renal findings, elevated serum IgG4, and IgG4-positive plasma cell infiltration in a hilar lymph node biopsy, supporting the diagnosis of systemic IgG4-RD

Twenty months post-hepatectomy, the patient remained free of colorectal cancer recurrence and showed no clinical or radiological evidence of IgG4-RD progression. After multidisciplinary discussion, neither adjuvant chemotherapy nor steroid therapy was initiated, and the patient continued under observation without treatment. The overall clinical course is summarized in Fig. 7.

## Discussion

In the present case, the S7 lesion showed necrotic adenocarcinoma cells with ghost-like morphology, surrounded by fibrotic tissue and chronic inflammatory cell infiltration, with prominent accumulation of IgG4-positive plasma cells. The necrotic tumor cells exhibited weak cytoplasmic positivity for AE1/AE3 and CDX2 without nuclear staining, confirming their nonviability. These findings suggest possible spontaneous regression of CRLM in the S7 lesion, accompanied by IgG4-related lymphoplasmacytic-type IPT-like inflammatory changes.

Spontaneous regression of cancer is extremely rare, and in CRLM, including the present case, only seven cases have been reported to date (Table 2) [8–13]. The mechanisms underlying tumor regression remain uncertain; however, a combination of factors, including alterations in the tumor microenvironment, host immune activation, ischemia, and tissue necrosis, has been proposed [3, 13]. Several reports have described the spontaneous regression of colorectal cancer or its metastases following immune status modulation. For instance, Pau et al. reported the regression of peritoneal metastases after methotrexate withdrawal in a patient with rheumatoid arthritis, attributing it to immune reactivation [14]. Zwart et al. observed regression of multiple CRLMs in a lung transplant recipient with mismatch repair–deficient colorectal cancer after cessation of immunosuppressive therapy, suggesting the reconstitution of immune surveillance [13]. Matsuki et al. described a case of postoperative regression of CRLM associated with chronic biliary infection, possibly attributable to local immune stimulation [11]. Collectively, these cases indicate that immunological factors may play a role in the spontaneous regression of CRLM.

**Table 2:** Clinicopathological features of reported spontaneous CRLM regression

Author Year	Age	Sex	Background of patient	Primary CRC: location, surgical procedure, and histopathological findings	Timing of CRLM onset, number, location, size	Diagnostic methods for CRLM	Diagnostic methods for spontaneous regression of CRLM	Histopathological evaluation methods, histological features of regressed CRLM	IgG4-positive plasma cell infiltration and/or IPT-like histological changes	Hypotheses for spontaneous regression of CRLM	Follow up period
Rankin [8] 1965	44	M	Familial polyposis	1st: Lower rectum, proctosigmoidectomy and combined APR, Grade 4 differentiated tubular adenocarcinoma 2nd: Ascending colon, Right colon resection, well-differentiated tubular adenocarcinoma	Synchronous at 1st CRC, 4 lesions, Right lobe	Biopsy at 1st CRC surgery: poorly differentiated carcinoma	Perioperative findings at 2nd operation	Not confirmed histologically.	Not mentioned	Not mentioned	10 years Alive
Francis [9] 1997	69	M	Unremarkable	Rectosigmoid junction, HAR, Moderate to poorly differentiated tubular adenocarcinoma	Synchronous, Multiple, Both lobes, Greater than 7 cm	Tru-cut biopsy at CRC surgery: fibrous tissue infiltrated with irregular clumps of neoplasm consistent with metastatic adenocarcinoma	US and CT showed regression of CRLM.	Not confirmed histologically.	Not mentioned	Removal of a source of trophic agents to an immunological effect	11 years Alive
Ikuta [10] 2002	60	M	Unremarkable	Lower rectum, APR and partial hepatectomy, Adenosquamous carcinoma (pT3pN3pM1)	Metachronous (4 months post primary tumor surgery), Mulitple, almost completely occuped both lobes	Follow-up CT	Follow-up CT showed remarkable regression of CRLM in the both lobes, autopsy	Autopsy, Chronic inflammation and coagulative necrosis with a few nodules of residual tumor.	Not mentioned	The biology of adenosquamous carcinoma or vascular obstruction due to enlarged hilar lymph nodes.	18 months Died due to peritoneal carcinomatosis.
Matsuki [11] 2018	72	F	Underwent PPPD for pancreatic head IPMN	Ascending colon, RHC, Moderately differentiated tubular adenocarcinoma (pT2pN1cM0)	Metachronous (5 months post primary tumor surgery), solitary, S3, 13 mm	CT, EOB-MRI, abdominal US	S3 segmentectomy	S3 Segmentectomy, Necrotic columnar epithelial cells surrounded by histiocytes, inflammatory cells and fibrosis.	Not mentioned	Host immune response to chronic biliary infection at the hepaticojejunostomy site after PPPD.	13 months Alive
Subasinghe [12] 2022	53	M	Unremarkable	Ascending colon, Lap-eRHC, Moderately differentiated tubular adenocarcinoma (pT2pN0cM0)	Metachronous (12 months post primary tumor surgery), solitary, S8, 21 mm	CT, EOB-MRI	S8 partial hepatectomy	S8 partial hepatectomy, Necrotic columnar epithelial cells surrounded by chronic inflammation and fibrosis.	Not mentioned	Infarct-like necrosis without chemotherapy, possibly induced by an immune-mediated injury following liver biopsy	12 months Alive
Zwart [13] 2023	59	F	Underment left lung transplantion for COPD, recurrent pulmonary infections under immunosuppression	Cecum, Biopsy, Mucinous adenocarcinoma	Synchronous, multiple, both lobes	CT, PET-CT, biopsy: adenocarcinoma (MLH1, PMS2, MSH6 loss; dMMR, BRAF V600E mutated, RAS wild type)	Follow-up CT showed remarkable regression of CRLM in the both lobes.	Autopsy, More than 40 CRLM lesions regressed completely, but the primary mucinous adenocarcinoma of the cecum did not.	Not mentioned	Withdrawal of immunosuppression activated anergic T cells, inducing regression of dMMR CRLM via anti-tumor immunity	7 months Died due to resrictive allograft syndrome
Our case	71	M	IgG4-RD	Rectum, Lap-Hartman’s operation, Moderately differentiated tubular adenocarcinoma (pT3pN0cM0)	Metachronous (8 months post primary tumor surgery), 2 lesions, S5 and S7, 6 mm and 11 mm	CT, EOB-MRI, PET-CT, enhance abdominal US	S5, S7 partial hepatectomy	S5, S7 partial hepatectomy, Necrotic CRLM surrounded by hepatic IPT, lymphoplasmacytic type	Marked IgG4-positive plasma cell infiltration with lymphoplasmacytic-type hepatic IPT-like changes	Intrahepatic presentation of CRLM antigens triggered an immune response, leading to tumor necrosis via IgG4-positive plasma cells, fibrosis, cytokines, and vascular damage.	20 months Alive

CRC, colorectal cancer; CRLM, colorectal liver metastasis; APR, abdominoperineal resection; HAR, high anterior resection; RHC, right hemicolectomy; Lap-eRHC, laparoscopic extended right hemicolectomy; PPPD, pylorus-preserving pancreaticoduodenectomy; IPMN, intraductal papillary mucinous neoplasm; COPD, chronic obstructive pulmonary disease; dMMR, deficient mismatch repair; IgG4-RD, immunoglobulin G4–related disease, IPT, inflammatory pseudotumor.

In the present case, abundant IgG4-positive plasma cells were observed around the regressed S7 lesion, along with storiform fibrosis and obliterative phlebitis, which are histological features compatible with the spectrum of IgG4-related hepatic IPT. Additionally, markedly elevated serum IgG4 levels, diffuse pancreatic enlargement with mild main pancreatic duct dilatation, multiple renal low-attenuation areas with mild cortical atrophy, and IgG4-positive plasma cell infiltration in mediastinal lymph nodes obtained via TBNA fulfilled the comprehensive diagnostic criteria for IgG4-RD. Considering these systemic findings, the accumulation of IgG4-positive plasma cells surrounding the regressed S7 metastatic lesion may reflect an IgG4-RD–related immune response rather than a nonspecific inflammatory response. A direct causal relationship between IgG4-RD and tumor regression could not be established. Nonetheless, their coexistence in the present case suggested the presence of a unique inflammatory environment at the metastatic site.

Hepatic IPTs are rare, heterogeneous entities with diverse etiologies, including autoimmune conditions and infections [15]. The lymphoplasmacytic subtype, which belongs to the IgG4-RD spectrum, is histologically characterized by dense lymphoplasmacytic infiltration, storiform fibrosis, and obliterative phlebitis [4, 16]. Most IgG4-related hepatic IPTs occur in the perihilar region and are often associated with IgG4-related sclerosing cholangitis [17]. Conversely, the lesion in this case was located in the peripheral parenchyma, a relatively uncommon site accounting for < 3% of the reported cases [18]. The presence of necrotic adenocarcinoma remnants within an IgG4-hepatic IPT–like lesion is unusual and expands the histological spectrum of hepatic IPTs associated with malignancy.

Recent studies have reported cases of “tumor-associated IgG4-RD,” in which IgG4-RD develops shortly after cancer diagnosis, raising the possibility of an immunological association between malignancy and IgG4-RD [16, 19]. Large cohort analyses have shown an increased prevalence of malignancy among patients with IgG4-RD compared with the general population, although the underlying mechanisms remain unclear [16]. Several mechanisms have been proposed to explain this association, including: (1) aberrant immune responses to tumor-associated self-antigens that trigger IgG4-RD; (2) shared inflammatory or genetic pathways predisposing to cancer and IgG4-RD; and (3) therapy-induced immune activation following tumor destruction, which may release tumor peptides and elicit secondary autoimmune responses in non-neoplastic tissues [5]. However, a definitive causal or histopathological link between malignancy and IgG4-RD has not been established.

Furthermore, recent immunopathological insights suggest that chronic inflammation, IgG4-positive plasma cell infiltration, and the activity of T-follicular helper cells, M2 macrophages, and plasmacytoid dendritic cells contribute to tumor–immune modulation in IgG4-RD [19]. However, previous studies have demonstrated that IgG4 antibodies may impair effective antitumor immunity by inhibiting IgG1-mediated effector functions, such as antibody-dependent cellular cytotoxicity, through interference with Fc receptor signaling [20]. Collectively, these findings support a model of complex bidirectional interactions between malignancy and IgG4-related immune dysregulation. In this context, the IgG4-rich inflammatory response observed in the present case may represent an immunomodulatory microenvironment with tumor-promoting and tumor-suppressive potential rather than a direct driver of tumor regression, although a contributory role cannot be entirely excluded.

In the present case, the possibility of spontaneous tumor regression should be limited to the S7 lesion, in which necrotic tumor structures were identified histologically. This lesion also showed accompanying IgG4-rich IPT-like inflammatory changes, suggesting that both processes may have occurred concurrently within the same lesion. The diagnosis of hepatic IPT prompted further systemic evaluation, which revealed elevated serum IgG4 levels and IgG4-positive plasma cell infiltration in distant lymph nodes, fulfilling the diagnostic criteria for IgG4-RD. Pre-hepatectomy imaging findings were indistinguishable from those of typical CRLM; therefore, surgical resection was an appropriate diagnostic and therapeutic approach. Histopathologic examination of the resected specimens provided findings suggestive of tumor regression in the S7 lesion.

To our knowledge, this is the first documented case of spontaneous CRLM regression accompanied by IgG4-related hepatic IPT–like changes. Although the underlying mechanism remains uncertain, the coexistence of tumor regression and IgG4-related inflammation in this case suggests, but does not prove, that immune-mediated and microenvironmental factors may be relevant in CRLM biology. Because this report describes a single case and no detailed immunological analyses, such as lymphocyte subset profiling or cytokine evaluation, were performed, a causal relationship could not be established. Nevertheless, the accumulation of similar cases and further immunological investigations will be necessary to better understand the potential interplay between inflammatory responses, including IgG4-related reactions, and tumor regression.

In conclusion, this case represents a rare instance of possible pathologically spontaneous regression of CRLM with coexisting IgG4-rich IPT-like changes. The findings suggest coexistence of tumor regression and IgG4-related inflammatory changes within the same lesion, although a causal relationship between these processes remains uncertain. This case provides valuable insights into the potential interactions between tumor regression, hepatic IPTs, and host immune responses in CRLM.

## Abbreviations

AE1/AE3: Cytokeratin AE1/AE3
CA19-9: Cancer antigen 19-9
CEA: Carcinoembryonic antigen
CRLM: Colorectal liver metastasis
CT: Computed tomography
EBUS-TBNA: Endobronchial ultrasound-guided transbronchial needle aspiration
FDG: Fluorodeoxyglucose
HER2: Human epidermal growth factor receptor 2
HPF: High-power field
IgG: Immunoglobulin G
IgG4: Immunoglobulin G4
IgG4-RD: IgG4-related disease
IPT: Inflammatory pseudotumor
MRI: Magnetic resonance imaging
PET-CT: Positron emission tomography-computed tomography
R0: Microscopically margin-negative resection
RAS: Rat sarcoma viral oncogene homolog
TBNA: Transbronchial needle aspiration
